# Clinical characteristics and predictors of survival in 284 patients with recurrent epithelial ovarian cancer: a large-scale retrospective analysis

**DOI:** 10.1097/MS9.0000000000003297

**Published:** 2025-04-15

**Authors:** Shahrzad Sheikhhasani, Azam Sadat Moosavi, Setareh Akhavan, Narges Zamani, Elahe Rezayof, Mina Sadat Mosavat

**Affiliations:** a Department of Gynecology Oncology, Tehran University of Medical Sciences, Valie-E-Asr Hospital, Tehran, Iran; b Vali-E-Asr Reproductive Health Research Center, Family Health Research Institute, Tehran University of Medical Sciences, Tehran, Iran

**Keywords:** cytoreductive surgery, ovarian cancer, prognostic factors, recurrence, survival

## Abstract

**Background:**

This study aimed to analyze clinical characteristics, recurrence patterns, and survival predictors in patients with recurrent epithelial ovarian cancer.

**Methods:**

We conducted a retrospective analysis of 284 patients with recurrent epithelial ovarian cancer treated at our institution between 2012 and 2022. Clinical data, recurrence patterns, treatment modalities, and survival outcomes were evaluated. Kaplan–Meier analysis and Cox proportional hazards models were used to assess survival and identify prognostic factors.

**Results:**

The mean age at recurrence was 54.47 years. Multiple site recurrence (50.70%) was the most common, followed by peritoneal (21.47%) and distant metastases (15.84%). Median progression-free survival (PFS) and overall survival (OS) were 5.00 and 23.00 months, respectively. Retroperitoneal recurrence was associated with better survival compared to distant recurrence (median OS 29.00 vs 16.00 months, *P* = 0.007). Multivariate analysis identified residual disease (HR 2.15, *P* < 0.001), elevated CA-125 (HR 1.6, *P* = 0.02), high-grade histology (HR 1.5, *P* = 0.01), and advanced initial stage (HR 1.7, *P* = 0.002) as significant predictors of poor survival.

**Conclusion:**

Recurrence patterns and timing significantly impact survival in recurrent ovarian cancer. Complete cytoreduction, tumor grade, and initial stage are crucial prognostic factors. These findings emphasize the need for personalized treatment strategies and continued research into novel therapeutic approaches.

## Introduction

Ovarian cancer remains a formidable gynecological malignancy due to its late presentation and high recurrence rate^[^1,2^]^. Over 70% of women are diagnosed at an advanced stage, compromising treatment options and long-term survival^[^3^]^. The alarmingly high recurrence rate, exceeding 60% after initial treatment, significantly impacts prognosis and quality of life^[^4^]^.

Recurrent ovarian cancer poses a critical challenge, often exhibiting resistance to therapy and poorer outcomes. Extensive research has explored factors influencing recurrence, including the extent of cytoreduction surgery, tumor histology, and subsequent treatment efficacy^[^5^]^. However, comprehensive analyses of long-term outcomes and recurrence patterns remain limited. The need for ongoing surveillance and personalized treatment strategies for effectively managing recurrent ovarian cancer has been well-emphasized^[^6,7^]^.

**HIGHLIGHTS**

- Multiple site recurrence (50.70%) was the most common pattern in recurrent epithelial ovarian cancer, followed by peritoneal (21.47%) and distant metastases (15.84%).
- Retroperitoneal recurrence was associated with better survival than distant recurrence (median OS 29.00 vs 16.00 months, *P* = 0.007).
- Residual disease after surgery was the strongest predictor of poor survival (HR 2.15, *P* < 0.001) in patients with recurrent epithelial ovarian cancer.
- High-grade histology, advanced initial stage, and elevated CA-125 levels were identified as significant predictors of poor survival in recurrent ovarian cancer.

This study aims to bridge this knowledge gap by providing a detailed analysis of the prognosis and outcomes in patients with recurrent ovarian cancer treated at our tertiary hospital over ten years (2012–2022). We endeavor to answer the following critical questions: (1) What are the primary factors influencing the recurrence of ovarian cancer in patients initially treated at our center? (2) How do different patterns and timing of recurrence impact patient survival and treatment outcomes? (3) What are the effective strategies employed to manage and improve prognosis in patients with recurrent ovarian cancer?

## Materials and methods

This study is a retrospective cohort analysis approved by the Institutional Review Board of our hospital (IR.TUMS.IKHC.REC.1402.320). It focused on the treatment outcomes of patients diagnosed with epithelial ovarian cancer from 1 January 2012, through 31 December 2022. This research adheres to the STROCSS criteria for reporting^[^8^]^. Eligible patients were identified from our institutional tumor registry database. Inclusion criteria encompassed histologically confirmed epithelial ovarian cancer with platinum-sensitive recurrence, defined as disease recurrence ≥6 months after completion of primary platinum-based chemotherapy. Patients with non-epithelial ovarian malignancies, platinum-resistant recurrence, or incomplete medical records were excluded. The final cohort comprised 284 patients with platinum-sensitive recurrent epithelial ovarian cancer who experienced disease recurrence during the study period and had complete clinical and follow-up data available for analysis.

Eligibility criteria for study inclusion required the following: (1) primary treatment received at our center, (2) documented recurrence of ovarian cancer during the study period, and (3) ≥12 months between initial diagnosis and recurrence. Patients were excluded if they had concurrent malignancies, insufficient follow-up data, or non-epithelial ovarian cancers.

All women had recurrent ovarian carcinoma diagnosed or confirmed by radiographic imaging studies, which consisted of a computed tomography (CT) scan, positron emission tomography (PET) scan, magnetic resonance image (MRI), or a combination. Data were meticulously collected from electronic medical records by trained research assistants (S.S.H.) and independently verified by two gynecologic oncologists.

The following information was gathered for each patient: Demographic information (age at diagnosis, menopausal status, BMI, comorbidities), Clinical characteristics at initial diagnosis (FIGO stage^[^9^]^, histological subtype, tumor grade, presence of ascites, serum CA-125 and CA-19-9 levels), Initial treatment details (type of primary surgery, extent of cytoreduction, chemotherapy regimens), Recurrence information (date of recurrence, time to recurrence, method of detection, pattern of recurrence, sites of recurrence), Treatment of recurrence (secondary cytoreductive surgery, second-line chemotherapy, response to treatment), Follow-up data (progression-free survival, overall survival, date of last follow-up or death). Gynecologic pathologists assessed all surgical specimens at our institution.

The primary outcome measure was progression-free survival (PFS), defined as the time from the start of treatment for recurrent disease until disease progression or death from any cause, whichever occurred first. Disease progression was determined using Response Evaluation Criteria in Solid Tumors (RECIST) version 1.1^[^10^]^. Overall survival (OS) was defined as the time from the date of recurrence diagnosis to the date of death from any cause or last follow-up.

Treatment response in recurrent disease was evaluated using RECIST 1.1 criteria. Responses were categorized as Complete Response (CR: the disappearance of all target lesions), Partial Response (PR: ≥ 30% decrease in the sum of target lesion diameters), Stable Disease (SD: neither PR nor PD criteria met), or Progressive Disease (PD: ≥ 20% increase in the sum of target lesion diameters). Response assessments were performed every 8–12 weeks or as clinically indicated, using radiological evaluation and serum CA-125 levels.

Recurrence was defined as the re-emergence of disease following a period of remission, confirmed by one or more of the following criteria:^[^1^]^ manifestation of clinical symptoms consistent with disease progression;^[^2^]^ elevation of serum CA-125 levels exceeding 35 U/mL, with a minimum doubling from the upper limit of normal; or^[^3^]^ radiological evidence of new lesions as per Response Evaluation Criteria in Solid Tumors (RECIST) version 1.1.

Recurrence patterns were systematically classified into four distinct categories: (A) Retroperitoneal: Confined to lymphatic spread in the retroperitoneal region, including pelvic and para-aortic lymph nodes. (B) Peritoneal: Characterized by transcoelomic spread within the peritoneal cavity, including pelvic and abdominal dissemination. (C) Mixed: A combination of retroperitoneal and peritoneal involvement indicates a more extensive disease spread. (D) Distant: Metastatic lesions beyond the peritoneal cavity, including but not limited to hepatic, pulmonary, osseous, or cerebral involvement, suggestive of hematogenous dissemination.

We employed the Kaplan–Meier method to estimate progression-free survival (PFS) and overall survival (OS). Survival curves between groups were compared using log-rank tests. Univariate and multivariate Cox proportional hazards models were applied to identify significant prognostic factors affecting survival outcomes (progression-free survival and overall survival). Hazard ratios (HR) and 95% confidence intervals (CI) were calculated. Cross-tabulations were analyzed with chi-square or Fisher exact tests, as appropriate. Statistical analyses were conducted using SPSS software (version 27.0, IBM Corp.) and SAS software packages. Confidence intervals were set at the 95% level, and *P* values were two-sided with a significance threshold of <0.05. To ensure robustness, sensitivity analyses were performed by excluding patients with borderline tumors and by stratifying patients based on the type of primary treatment (primary debulking surgery vs. neoadjuvant chemotherapy followed by interval debulking surgery).

## Results:

### Patient characteristics and preoperative imaging studies

From 510 patients initially diagnosed with epithelial ovarian cancer at our institution, 332 experienced recurrences. After applying exclusion criteria, we analyzed 284 cases. The average age at diagnosis was 52.73 ± 11.38 years (range: 29–73 years), and at recurrence, it was 54.47 ± 11.17 years (range: 32–74 years). Most patients (61.61%) were postmenopausal at diagnosis. Table 1 provides a summary of demographic and clinical characteristics.

**Table 1:** Patient demographics and clinical characteristics

Characteristic	Mean (Range) or n (%)
Age at diagnosis (years)	52.73 (29–73)
Age at recurrence (years)	54.47 (32–74)
Menopausal status	
- Premenopausal	109 (38.38%)
- Postmenopausal	175 (61.61%)
BMI	26.5 (18.2–37.8)
Comorbidities	
- Hypertension	117 (41.97%)
- Diabetes	148 (52.11%)
- Cardiovascular disease	100 (35.21%)
- Other	19 (6.69%)
Tumor grade	
- Low grade	61 (21.47%)
- High grade	223 (78.52%)
Presence of ascites	202 (71.12%)
Serum CA-125 at diagnosis (U/mL)	575.40 (48–1200)

Comorbidities were present in 45.77% of patients, with hypertension being the most prevalent (41.97%). Most tumors were high-grade (78.52%). Ascites were present in 71.12% of patients at diagnosis. The mean serum CA-125 level was 575.40 U/mL (48–1200 U/mL). The most common histological subtype was serous carcinoma (89.08%), followed by clear cell (6.33%), mucinous (2.81%), and endometrioid (1.76%) carcinomas. A majority presented with advanced-stage disease: 83.09% were FIGO stage III, and 4.92% were stage IV.

Primary debulking surgery followed by chemotherapy was administered to 57.04% of patients, while 42.96% received neoadjuvant chemotherapy followed by interval debulking surgery.

### Patterns and timing of recurrence

The average time to recurrence was 19.83 ± 19.37 months. Recurrence was detected via elevated CA-125 levels (54.76%), clinical symptoms (35.71%), and radiological evidence (9.52%). Recurrence patterns included: Retroperitoneal (lymph node): 34 (11.97%), Peritoneal: 61 (21.47%), Multiple (retroperitoneal + peritoneal): 144 (50.70%), Distant (hepatic, splenic, bone, cerebral, adrenal, kidney, skin): 45 (15.84%).

### Secondary treatment and survival outcomes

Secondary cytoreductive surgery was performed in 14.43% of patients; 85.56% received second-line chemotherapy. Common regimens included Taxol + carboplatin (78.17%), Taxol + carboplatin + bevacizumab (14.79%), and others(7.04%).

**Figure 1.**
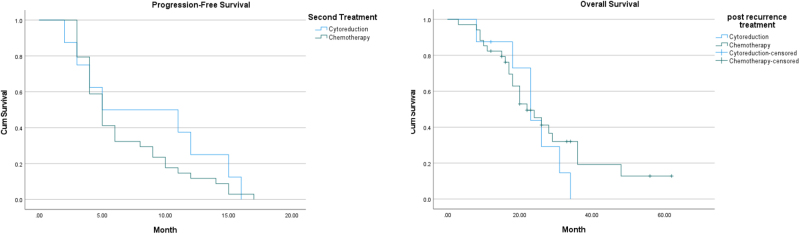
Kaplan–Meier survival curves for patients with recurrent epithelial ovarian cancer. (A) Progression-free survival (PFS) curve showing a median PFS of 7.02 months (SE: 0.68). (B) Overall survival (OS) curve demonstrating a median OS of 26.63 months (SE: 2.6).

The median progression-free survival (PFS) post-recurrence was 5.00 months (SE: 0.46), with an overall survival (OS) of 23.00 months (SE: 2.73; Table 2; Fig. 1).

**Table 2:** Treatment modalities and survival outcomes

Treatment modality	Frequency (%)	Median PFS (months)	Median OS (months)
Secondary cytoreductive surgery	41 (14.43%)	5.00 ± 4.95	23.00 ± 3.22
Second-line chemotherapy	243(85.56%)	5.00 ± 0.47	22.00 ± 3.31

### Factors influencing recurrence and survival

Patients with a recurrence interval of 18 months or more showed improved survival outcomes, with a median OS of 23.0 ± 1.65 months compared to 20.0 ± 5.56 months for intervals shorter than 18 months (*P* = 0.121). Recurrence pattern significantly affected survival (*P* = 0.007). Retroperitoneal recurrence correlated with better survival (median OS 29.00 ± 1.30 months) than distant recurrence (median OS 16.00 ± 3.38 months; Table 3).

**Table 3:** Impact of recurrence patterns and timing on survival

Factor	Subcategory	HR (OS) (Months)	95% CI	*P* value
Time to recurrence	<18 months	Ref		0.121
	≥18 months	0.89	0.79–1.05	
Pattern of recurrence	Retroperitoneal	Ref		0.007
	Peritoneal	1.46	1.19–1.88	
	Mix	1.61	1.33–1.91	
	Distant	2.4	1.91–3.1	

### Multivariate analysis of predictors of survival

Using multivariate Cox proportional hazards models, we identified several independent prognostic factors for survival in recurrent epithelial ovarian cancer. Residual disease following suboptimal cytoreduction was the strongest predictor of poor overall survival (HR 2.15, 95% CI: 1.5–3.1, *P* < 0.001), emphasizing the critical role of complete tumor resection in improving patient outcomes. Additionally, elevated CA-125 levels (≥100 U/mL) were significantly associated with shorter survival (HR 1.6, 95% CI: 1.1–2.4, *P* = 0.02), reinforcing the prognostic value of this biomarker in disease monitoring.

Tumor histological grade and initial FIGO stage were also significant predictors. Patients with high-grade tumors had an increased risk of mortality (HR 1.5, 95% CI: 1.1–2.1, *P* = 0.01), while those with FIGO stage III/IV disease at initial diagnosis had worse overall survival (HR 1.7, 95% CI: 1.2–2.4, *P* = 0.002), indicating that tumor biology and disease burden strongly impact recurrence and survival outcomes.

Conversely, age at recurrence (≥60 years) (HR 1.4, 95% CI: 0.91–1.77, *P* = 0.223) and diagnosis-to-recurrence interval (<18 months) (HR 0.991, 95% CI: 0.958–1.025, *P* = 0.581) were not significantly associated with survival. These findings suggest that intrinsic tumor characteristics and response to treatment may have a greater prognostic influence than patient age or early recurrence timing.

Table 4 summarizes these findings, presenting hazard ratios (HR) and 95% confidence intervals (CI) for each variable analyzed. These results highlight the importance of surgical outcomes, tumor burden, and biomarker surveillance in guiding the management and prognosis of patients with recurrent epithelial ovarian cancer.

**Table 4:** Multivariate analysis of predictors of survival

Predictor	Subcategory	Hazard ratio (HR)	95% confidence interval (CI)	*P* value
Residual disease	Suboptimal surgery	2.15	1.5–3.1	<0.001
Diagnosis-to-recurrence interval	<18 months	0.991	0.958–1.025	0.581
Serum CA-125	≥100 U/mL	1.6	1.1–2.4	0.02
Age at recurrence	≥60 years	1.4	0.91–1.77	0.223
Histological grade	High Grade	1.5	1.1–2.1	0.01
Initial FIGO stage	III/IV	1.7	1.2–2.4	0.002

## Discussion

This large-scale retrospective analysis of 284 patients with recurrent epithelial ovarian cancer provides valuable insights into the clinical characteristics, patterns of recurrence, and factors influencing survival in this challenging patient population. Our findings contribute to the growing body of knowledge on recurrent ovarian cancer management and highlight areas for future research.

Our study revealed diverse patterns of recurrence, with multiple site involvement (retroperitoneal + peritoneal) being the most common (50.70%), followed by isolated peritoneal (21.47%) and distant metastases (15.84%). This distribution differs somewhat from previous reports, such as the study by Petrillo *et al*^[^11^]^, which found isolated peritoneal recurrence more frequent (60.7%). Our overall recurrence rate exceeds 60%, which aligns with previously published retrospective studies examining long-term outcomes in epithelial ovarian cancer^[^4,12^]^. Several factors may explain this high recurrence rate in our cohort. First, our institution is a national referral center, meaning we receive a higher proportion of advanced-stage and high-risk patients, which inherently increases recurrence likelihood. Second, the majority of our cohort presented with FIGO stage III/IV disease, which is well documented as a predictor of poor prognosis and higher relapse rates. Third, we exclusively included platinum-sensitive recurrent ovarian cancer cases, which may have introduced a selection bias by capturing patients who had already relapsed post-treatment. Recognizing these factors is essential to understanding our results and their clinical implications for patient management and long-term surveillance.

Our study’s correlation between recurrence patterns and survival outcomes aligns with previous research and offers new insights. Retroperitoneal recurrence was associated with better survival (median OS 29.00 months) than distant recurrence (Median OS 16.00 months). This finding is consistent with the work of Ferrero *et al*^[^12^]^, who reported improved outcomes for isolated lymph node recurrence compared to peritoneal recurrence. The prognostic significance of recurrence localization underscores the importance of thorough imaging and careful evaluation of recurrence patterns in clinical decision-making.

While not reaching statistical significance in our study (*P* = 0.121), the impact of recurrence timing on survival aligns with the established concept of platinum sensitivity in ovarian cancer, as reported in previous studies by Angeles *et al* and Hogen *et al*^[^13,14^]^. Patients with a recurrence interval of 18 months or more showed a trend towards improved survival outcomes. This observation is consistent with the work of Paik *et al*, who demonstrated significantly better overall survival in patients with longer platinum-free intervals^[^15^]^. Despite not reaching statistical significance, the trend observed in our study supports the continued use of the platinum-free interval as a key factor in treatment decision-making and prognostication.

Our multivariate analysis identified several significant predictors of poor survival, providing valuable insights into the complex interplay of factors affecting patient outcomes in recurrent ovarian cancer. Residual disease after primary surgery emerged as a strong predictor of poor survival (HR 2.15, *P* < 0.001), reinforcing the critical importance of complete cytoreduction. This finding aligns with the meta-analysis by Bristow *et al* and Baek *et al*, which demonstrated a clear correlation between the extent of cytoreduction and survival outcomes^[^16,17^]^. Elevated serum CA-125 levels ≥100 U/mL at recurrence were associated with poorer survival outcomes (HR 1.6, *P* = 0.02), consistent with the literature^[^18,19^]^. This result supports the continued use of CA-125 as a valuable biomarker in monitoring disease status and predicting outcomes in recurrent ovarian cancer. However, it is important to note that CA-125 levels should be interpreted with other clinical and radiological findings, as emphasized by recent guidelines^[^20,21^]^. High-grade histology (HR 1.5, *P* = 0.01) and initial FIGO stage III/IV (HR 1.7, *P* = 0.002) were identified as significant predictors of poor survival, consistent with the established understanding of ovarian cancer biology and natural history. These findings underscore the importance of considering tumor grade and initial stage in risk stratification and treatment planning for patients with recurrent ovarian cancer.

The management approaches observed in our cohort reflect the evolving landscape of recurrent ovarian cancer treatment. The current gold standard of care for recurrent epithelial ovarian cancer includes complete cytoreductive surgery when feasible, combined with platinum-based chemotherapy, as recommended by leading guidelines. In our study, 85.56% of patients received second-line chemotherapy, while only 14.43% underwent secondary cytoreductive surgery. This surgical rate is lower than expected, which may be attributed to stringent selection criteria or advanced disease burden in our cohort.

Compared to previous studies, where optimal secondary cytoreduction has been shown to improve survival in carefully selected patients, our findings suggest that real-world application of this approach remains limited by disease extent, patient performance status, and institutional treatment protocols. These findings align with those reported by Fotopoulou *et al*^[^22^]^, highlighting the ongoing debate regarding the optimal management strategy for recurrent disease. Additionally, emerging targeted therapies and novel combination regimens may challenge the traditional reliance on cytoreductive surgery plus platinum-based chemotherapy, further shifting treatment paradigms in recurrent ovarian cancer.

The median progression-free survival (PFS) of 5.00 months and overall survival (OS) of 23.00 months observed in our study are comparable to those reported in recent clinical trials, such as the OCEANS study^[^23^]^. These outcomes underscore the persistent challenges in achieving long-term disease control in recurrent ovarian cancer and emphasize the need for continued research into novel therapeutic approaches. This observation aligns with the results of the DESKTOP III trial, which demonstrated a significant PFS benefit for secondary surgery in selected patients^[^24^]^. However, the lack of a significant OS difference in our study highlights the complexity of the decision-making process regarding secondary surgery and the need for careful patient selection.

While our study provides valuable insights into clinical factors affecting outcomes in recurrent ovarian cancer, it is important to acknowledge the growing role of molecular profiling in guiding treatment decisions. The advent of PARP inhibitors has revolutionized the management of BRCA-mutated and HRD-positive ovarian cancers^[^25,26^]^. Future studies should aim to integrate molecular profiling data with clinical and pathological factors to develop more comprehensive predictive and prognostic models^[^27^]^. Although not reflected in our cohort, the emerging role of immunotherapy in ovarian cancer represents an exciting avenue for future research^[^28^]^. Ongoing trials exploring combination strategies and biomarker-driven approaches may unlock the potential of immunotherapy in this challenging disease^[^29^]^.

Our study has several limitations that should be acknowledged. First, as a retrospective analysis, it is subject to inherent biases, including selection bias and potential confounding factors. The single-center nature of our study may limit the generalizability of our findings to other populations or healthcare settings. Additionally, the lack of data on important molecular markers, such as BRCA status and HRD, represents a significant limitation in the current era of precision oncology. The relatively small sample size in specific subgroups, particularly for secondary cytoreductive surgery, may have limited our ability to detect statistically significant outcome differences. Furthermore, changes in treatment paradigms and the introduction of novel therapies over the study period may have influenced outcomes in ways that are difficult to account for in a retrospective analysis.

## Conclusion

In conclusion, our comprehensive analysis of recurrent ovarian cancer patterns and outcomes provides valuable insights into the complex interplay of factors affecting prognosis and treatment response. The findings underscore the importance of recurrence pattern, timing, and complete cytoreduction in determining outcomes. While secondary cytoreductive surgery may benefit selected patients, the optimal management strategy remains individualized.

As the treatment landscape for recurrent ovarian cancer continues to evolve, a multidisciplinary approach considering patient factors, disease characteristics, and emerging therapies will be crucial. Future prospective studies should focus on integrating molecular profiling; novel targeted agents, and immunotherapies to personalize further and optimize the management of recurrent ovarian cancer. Additionally, efforts to develop and validate predictive models incorporating clinical, pathological, and molecular factors could significantly enhance our ability to tailor treatment approaches and improve outcomes for women with this challenging disease.

## Data Availability

The datasets generated and/or analyzed during the current study are not publicly available due to privacy concerns. However, they are available from the corresponding author, Dr. Mina Sadat Mosavat, on reasonable request.
